# Caspase 3‐specific cleavage of MEK1 suppresses ERK signaling and sensitizes cells to stress‐induced apoptosis

**DOI:** 10.1002/2211-5463.13574

**Published:** 2023-02-23

**Authors:** Hisashi Moriizumi, Yuji Kubota, Tomoyuki Tsuchiya, Ryosuke Naka, Mutsuhiro Takekawa

**Affiliations:** ^1^ Division of Cell Signaling and Molecular Medicine, Institute of Medical Science The University of Tokyo Japan; ^2^ Department of Computational Biology and Medical Sciences, Graduate School of Frontier Sciences The University of Tokyo Chiba Japan

**Keywords:** apoptosis, caspase, ERK, MEK, RASopathy

## Abstract

Proper regulation of apoptotic cell death is crucial for normal development and homeostasis in multicellular organisms and is achieved by the balance between pro‐apoptotic processes, such as caspase activation, and pro‐survival signaling, such as extracellular signal‐regulated kinase (ERK) activation. However, the functional interplay between these opposing signaling pathways remains incompletely understood. Here, we identified MAPK/ERK kinase (MEK) 1, a central component of the ERK pathway, as a specific substrate for the executioner caspase‐3. During apoptosis, MEK1 is cleaved at an evolutionarily conserved Asp282 residue in the kinase domain, thereby losing its catalytic activity. Gene knockout experiments showed that MEK1 cleavage was mediated by caspase‐3, but not by the other executioner caspases, caspase‐6 or ‐7. Following exposure of cells to osmotic stress, elevated ERK activity gradually decreased, and this was accompanied by increased cleavage of MEK1. In contrast, the expression of a caspase‐uncleavable MEK1(D282N) mutant in cells maintained stress‐induced ERK activity and thereby attenuated apoptotic cell death. Thus, caspase‐3‐mediated, proteolytic inhibition of MEK1 sensitizes cells to apoptosis by suppressing pro‐survival ERK signaling. Furthermore, we found that a RASopathy‐associated MEK1(Y130C) mutation prevented this caspase‐3‐mediated proteolytic inactivation of MEK1 and efficiently protected cells from stress‐induced apoptosis. Our data reveal the functional crosstalk between ERK‐mediated cell survival and caspase‐mediated cell death pathways and suggest that its dysregulation by a disease‐associated MEK1 mutation is at least partly involved in the pathophysiology of congenital RASopathies.

## Graphical abstract

Proper regulation of apoptosis is essential for normal development and homeostasis. Here, we identified MEK1 as a specific substrate for caspase‐3 during apoptosis. Cleavage of MEK1 at Asp282 by caspase‐3 inhibits MEK1 activity and ERK signaling, thereby sensitizing cells to stress‐induced apoptosis. The RASopathy‐associated MEK1(Y130C) mutation disrupts this mechanism, suggesting that its dysregulation is involved in the pathophysiology of RASopathies.

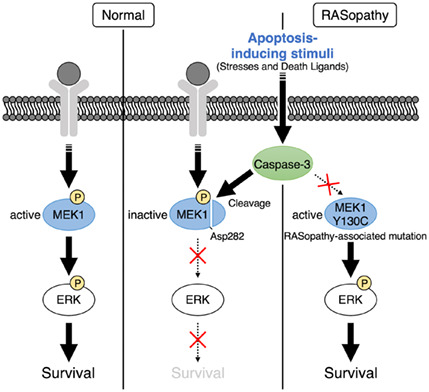

**Abbreviations**
ATP: adenosine triphosphate
CFC: cardio‐facio‐cutaneous
CRISPR: clustered regularly interspaced short palindromic repeats
dKO: double knockout
DTT: dithiothreitol
ERK: extracellular signal‐regulated kinase
GST: glutathione S‐transferase
KO: knockout
MAPK: mitogen‐activated protein kinase
MEFs: mouse embryonic fibroblasts
MEK: MAPK/ERK kinase
PARP: poly (ADP‐ribose) polymerase
PMSF: phenylmethylsulphonyl fluoride

Mammalian cells respond to extrinsic or intrinsic stresses in various ways, ranging from the activation of adaptive or reparative signaling to the initiation of cell death processes. Such protective and destructive signaling pathways are often interconnected, and their functional crosstalk plays a key role in the rigorous control of cell fate decisions under stress. While the induction of apoptotic cell death is a major destructive response to stress, a key cellular protective signaling pathway is the extracellular signal‐regulated kinase (ERK) cascade. The ERK pathway, which consists of a three‐tiered core of protein kinases (Raf, MEK, and ERK), is typically activated by growth factors and plays pivotal roles in a wide array of physiological and pathological processes, including cell proliferation, survival, embryonic development, and carcinogenesis [1]. In general, ERK signaling is initiated by activation of the Raf family kinases in a Ras‐dependent manner. Activated Raf then phosphorylates two conserved Ser residues within the T‐loops of MEK1/2, which in turn phosphorylate and activate ERK1/2. Upon activation, a portion of ERK1/2 translocates from the cytoplasm to the nucleus, and phosphorylates substrates, such as transcription factors (e.g., Elk1 and c‐fos), kinases (e.g., ribosomal S6 kinase [RSK] and MAP kinase‐interacting kinase [MNK]) and others. Through these consecutive phosphorylation events, ERK signaling regulates not only growth factor‐induced cellular processes, such as proliferation and motility, but also stress‐induced cell‐fate decisions, including survival and death. Indeed, the ERK pathway is activated by various stress stimuli (e.g., oxidative, osmotic, and genotoxic stress, and cytokines such as tumor necrosis factor‐α, and FasL) and growth factors [1, 2].

Although ERK signaling can occasionally contribute to the induction of cell growth arrest depending on the cell type and stimulus, previous studies demonstrated that its activation is generally associated with the suppression of apoptosis and promotes cell survival through several different mechanisms [3, 4]. For instance, ERK‐mediated phosphorylation of Bim, a Bcl‐2 homology‐3 domain (BH3)‐only pro‐apoptotic protein, impairs its interaction with the anti‐apoptotic protein, Bcl‐2, and facilitates ubiquitination‐dependent degradation of Bim, thereby preventing apoptosis [5, 6]. Phosphorylation of a transcription factor, FOXO3a, at S294/S344/S425 by ERK enhances its ubiquitin‐dependent degradation and inhibits the expression of its target pro‐apoptotic genes such as *FasL* and *Bim* [7]. Extracellular signal‐regulated kinase directly phosphorylates the initiator caspases, caspase‐8 (at S387) and caspase‐9 (at T125), and suppresses their activation [8, 9]. Furthermore, ERK‐mediated RSK activation induces phosphorylation of various pro‐apoptotic proteins, including Bad, DAPK, and Apaf‐1, and thereby curtails their cytotoxic activities [10, 11, 12]. In addition to the suppression of pro‐apoptotic proteins, ERK also enhances the expression and activity of anti‐apoptotic proteins, such as Mcl‐1 and IEX‐1, by phosphorylating these proteins [13, 14, 15]. Thus, ERK signaling exerts its cytoprotective effects on cells by downregulating pro‐apoptotic proteins and upregulating anti‐apoptotic factors through both transcriptional and posttranscriptional mechanisms.

MEK1 and MEK2, central components of the ERK cascade, are ubiquitously expressed and share a high level of amino acid sequence homology particularly in their kinase domains. However, they possess two regions of lower homology: (1) an N‐terminal region (amino acids 1–67 in MEK1), which contains an ERK‐docking site (also called a D‐site) and nuclear export sequence, and (2) a proline‐rich loop region (amino acids 262–307 in MEK1), which contributes to specific protein–protein interactions important for the regulation of the MEKs [16]. The observed differences in amino acid sequence between MEK1 and MEK2 imply that these proteins can have unique functions in cells. Indeed, previous studies using various cell lines and MEK1/2‐null mice have shown that these two kinases play overlapping and distinctive roles in the regulation of several biological processes, such as cell growth, survival, embryonic development, and carcinogenesis [17, 18, 19, 20, 21, 22]. However, the functional differences, if any, between MEK1 and MEK2 during apoptotic cell death remain unknown.

Besides their critical roles in physiological processes, MEK1/2 also serve as oncogenes in human cancer. Gain‐of‐function mutations of MEK1/2 are detected in various sporadic cancers, including melanoma, colorectal, lung, and ovarian cancers [23]. These MEK mutants hyperactivate ERK signaling and ultimately induce cancer development and progression. Moreover, recent clinical sequencing studies identified germline mutations of these genes in a group of congenital disorders termed RASopathies [24]. RASopathies, which include Costello, Noonan, cardio‐facio‐cutaneous (CFC), and Leopard syndromes, share many overlapping clinical features such as cranio‐facial dysmorphisms, neurocognitive impairment, cardiomyopathies, and cutaneous and musculoskeletal abnormalities. In particular, mutations in either MEK1 or MEK2 are detected in approximately 25% of individuals with CFC syndrome, and aberrantly enhance their kinase activities [23]. Despite the importance of MEK mutations in the etiology of cancer and RASopathies, the precise roles and regulation of disease‐associated MEK mutants during the apoptotic process remain totally unknown.

Apoptosis, a form of programmed cell death, is responsible for the removal of damaged or superfluous cells without releasing pro‐inflammatory cellular components and thus plays essential roles in the development and homeostasis of multicellular organisms [25]. Dysregulation of this cell death program is involved in a variety of human diseases, including developmental abnormalities, autoimmune diseases, neurodegenerative disorders, and cancer. The onset of apoptosis is mediated by at least two interrelated molecular processes: the extrinsic and intrinsic pathways [26]. The extrinsic pathway is initiated by the binding of specific death ligands (e.g., FasL) to their respective transmembrane death receptors. These receptors then recruit an adaptor FAS‐associated death‐domain protein and an initiator procaspase‐8 (or ‐10) to induce the assembly of the death‐inducing signaling complex and the subsequent activation of caspase‐8. In contrast, the intrinsic pathway is triggered in response to diverse cellular stresses (e.g., DNA damage and osmotic stress) and involves a Bcl‐2 family of proteins that regulate the release of pro‐apoptotic factors, including cytochrome *c*, from the mitochondria. Released cytochrome *c* binds to Apaf‐1 in the cytoplasm and promotes the formation of an apoptosome, which recruits and activates an initiator caspase, procaspase‐9. Both extrinsic and intrinsic pathways converge on the activation of executioner caspases (caspase‐3/6/7), and ultimately lead to apoptotic cell death. Although these executioner caspases cleave many proteins to regulate various cellular functions during apoptosis [27], their substrate specificity has yet to be fully defined.

Here, we identified MEK1 as a highly specific substrate for executioner caspase‐3. During apoptosis, MEK1 is efficiently cleaved at an Asp282 residue within its divergent proline‐rich region of the kinase domain, thereby losing its kinase activity. Analyses of cells deficient in various executioner caspases revealed that this MEK1 cleavage was mediated by caspase‐3, but not by caspase‐6 or ‐7. Cells expressing a caspase‐uncleavable MEK1(D282N) mutant exhibited increased ERK activity and decreased apoptosis under stress, as compared with cells expressing wild‐type MEK1. Thus, caspase‐3‐mediated MEK1 cleavage sensitizes cells to apoptosis by suppressing pro‐survival ERK signaling. We further showed that a RASopathy‐associated Y130C mutation renders MEK1 resistant to caspase‐dependent cleavage. Our results demonstrate the functional interplay between ERK‐mediated survival signaling and caspase‐mediated cell death signaling, and delineate a molecular mechanism that dictates cell‐fate decisions under stress. Because this mechanism is perturbed by the RASopathy‐associated MEK1 mutation, its dysregulation may be involved in the developmental abnormalities of congenital RASopathies.

## Materials and methods

### Plasmids

Flag‐MEK1, Myc‐MEK1, ERK2, Flag‐BRaf, and their derivative mutants were subcloned into pcDNA3 (Invitrogen, Waltham, MA, USA) or pQCXIP (Clontech, Mountain View, CA, USA). Constitutively active human MEK1(DD: S218D/S222D), rat ERK2(PD: L73P/S151D), and catalytically inactive rat ERK2(K/N: K52N) mutants were constructed using PCR‐based mutagenesis. pGEX‐6P (GE Healthcare, Chicago, IL, USA) and pCold (TaKaRa, Osaka, Japan) vectors were used for bacterial expression of glutathione S‐transferase (GST)‐tagged MEK1, MEK2, and caspase‐3.

### Media and buffers

Lysis buffer A contained 20 mm Tris–HCl (pH 7.5), 1% Triton X‐100, 0.5% deoxycholate, 10% glycerol, 137 mm NaCl, 2 mm EDTA, 50 mm β‐glycerophosphate, 10 mm NaF, 2 mm Na_3_VO_4_, 1 mm dithiothreitol (DTT), 1 mm phenylmethylsulphonyl fluoride (PMSF), 10 μg·mL^−1^ leupeptin, and 10 μg·mL^−1^ aprotinin. Lysis buffer B contained 20 mm Tris–HCl (pH 7.5), 1% Triton X‐100, 0.1% deoxycholate, 10% glycerol, 150 mm NaCl, 50 mm NaF, 1 mm DTT, 1 mm PMSF, 10 μg·mL^−1^ leupeptin, 10 μg·mL^−1^ aprotinin, and 100 nm microcystin‐LR. Kinase buffer contained 25 mm Tris–HCl (pH 7.5), 25 mm MgCl_2_, 10 mm β‐glycerophosphate, 2 mm Na_3_VO_4_, and 2 mm DTT. Cleavage assay buffer contained 25 mm Hepes (pH 7.5), 1 mm EDTA, 5 mm DTT, and 0.1% 3‐[(3‐cholamidopropyl) dimethylaminonio]‐1‐propanesulphonate (CHAPS).

### Cell culture and treatment

U2OS (HTB‐96), A375 (CRL‐1619), and H1299 (CRL‐5803) cell lines were obtained from the American Type Culture Collection (Manassas, VA, USA). Jurkat (RCB3052), HEK293 (RCB1637), A549 (RCB3677), and GP2‐293 (RCB2354) cell lines were obtained from RIKEN Cell Bank (Tsukuba, Japan). Plat‐E was provided by T. Kitamura (The University of Tokyo). *MEK1* ^−/−^ mouse embryonic fibroblasts (MEFs) were provided by J. Charron (Université Laval, Québec). Cells were maintained in Dulbecco's Modified Eagle Medium or RPMI1640 supplemented with 10% fetal bovine serum, l‐glutamate, penicillin, and streptomycin. Where indicated, the cells were stimulated with 60 μm etoposide (Sigma‐Aldrich, St Louis, MO, USA), 200 ng·mL^−1^ anti‐Fas antibody (MBL Life Science, Tokyo, Japan, clone CH‐11), or osmotic stress (sorbitol, 0.6 m) for the indicated times, unless otherwise noted. Where indicated, cells were pretreated with a pan‐caspase inhibitor [100 μm Z‐VAD‐FMK (Santa Cruz, Dallas, TX, USA)], a caspase‐8 inhibitor [20 μm Z‐IETD‐FMK (R&D Systems, Minneapolis, MN, USA, FMK007)], or a caspase‐9 inhibitor [20 μm Z‐LEHD‐FMK (R&D Systems, FMK008)] for 2 h, or with a MEK inhibitor [10 μm Trametinib (LC Laboratories, T‐8123)] for 30 min before stimulation.

### Transient transfection and generation of stable cell lines

For transient transfection, preseeded cells were transfected with appropriate expression plasmids using X‐tremeGENE 9 (Sigma‐Aldrich) according to the manufacturer's protocol. Stable cell lines derived from HEK293, U2OS, and MEF cells were generated by retroviral infection. Briefly, retroviruses were produced in GP2‐293 packaging cells by transient transfection with pVSV and pQCXIP plasmids. Culture supernatants were collected 48‐h post‐transfection, filtered, and supplemented with 8 μg·mL^−1^ polybrene. Cells were infected with retrovirus and selected with puromycin. Retroviral infection of MEFs was performed as described above, except that Plat‐E packaging cells were used.

### Generation of knockout cell lines

To construct clustered regularly interspaced short palindromic repeats (CRISPR)‐Cas9 plasmids targeting human CASP3, CASP6, or CASP7, target genomic DNA sequences were designed using an online design tool, CRISPRdirect (https://crispr.dbcls.jp). The following single guide (sg)RNA primers were used: CASP3 Forward, 5′‐CACCGCATACATGGAAGCGAATCAA‐3′; CASP3 Reverse, 5′‐AAACTTGATTCGCTTCCATGTATGC‐3′; CASP6 Forward, 5′‐CACCGCTCGGCCTCGGGGCTCCGCA‐3′; CASP6 Reverse, 5′‐AAACTGCGGAGCCCCGAGGCCGAGC‐3′; CASP7 Forward, 5′‐CACCGAAGAGGGACGGTACAAACG‐3′; CASP7 Reverse, 5′‐AAACCGTTTGTACCGTCCCTCTTC‐3′. Single‐guide RNA was prepared and inserted into a pSpCas9(BB)‐2A‐Puro expression plasmid. U2OS cells cultured in 35‐mm dishes were transfected with the appropriate expression plasmids using X‐tremeGENE9 (Sigma‐Aldrich). Puromycin‐selected cells were diluted and subcloned in 96‐well plates to collect single‐cell clones.

### Purification of recombinant proteins

The *Escherichia coli* strain, DH5α, was transformed with a pGEX‐6P‐based plasmid encoding GST‐ERK2(K52N), GST‐MEK1(WT or D282N), or GST‐MEK2. Exponentially growing DH5α cells carrying one of these plasmids were incubated with 0.5 mm isopropylthio‐β‐galactosidase (IPTG) at 25 °C for 14 h before harvesting. DH5α cells carrying a pCold‐based plasmid encoding caspase‐3‐GST were incubated with 0.5 mm IPTG at 15 °C for 24 h. Cells were suspended in cold phosphate‐buffered saline, lysed by sonication, and then clarified by centrifugation (30 000 ***g*** for 15 min at 4 °C). The clear supernatant was filtered through a 0.45 μm filter, and GST‐tagged proteins were purified using glutathione sepharose beads (Cytiva, Marlborough, MA, USA) and used for *in vitro* assays. Where indicated, the GST‐tag of purified GST‐MEK1 and GST‐MEK2 was removed by incubating with PreScission Protease (Cytiva) at 4 °C for 24 h in buffer containing 50 mm Tris–HCl (pH 7), 150 mm NaCl, 1 mm EDTA, and 1 mm DTT.

### *In vitro* caspase‐3 cleavage assay

An *in vitro* cleavage assay of recombinant caspase‐3 was performed as described [28]. Briefly, recombinant MEK1 or MEK2 proteins (0.6 μg) were incubated with caspase‐3‐GST (0.6 μg) at 37 °C in cleavage assay buffer. Proteins in the reaction mixtures were separated by sodium dodecyl sulfate polyacrylamide gel electrophoresis (SDS/PAGE) and subjected to immunoblotting.

### *In vitro* kinase assay

Recombinant MEK1 proteins (0.6 μg) were mixed with inactive ERK2(K52N) (0.6 μg) in kinase buffer (40 μL) containing 160 μm ATP, and were incubated at 25 °C. Phosphorylated ERK in the reaction samples was analyzed by immunoblotting using a phospho‐specific ERK antibody (Cell Signaling Technology, Danvers, MA, USA, 9101). For an *in vitro* kinase assay shown in Fig. 5A, recombinant MEK1 protein digested with caspase‐3 at 37 °C was used.

### Immunoblotting analysis

For immunoblot analysis, cells were lysed in lysis buffer A, except when immunoprecipitation and Phos‐tag SDS/PAGE were performed, as described below. Digitized images were captured by ImageQuant LAS4000 (GE Healthcare). The following primary antibodies were used: monoclonal anti‐GST B‐14 (Santa Cruz, sc‐138), anti–Myc 9E10 (Santa Cruz, sc‐40), anti‐ERK1/2 C‐9 (Santa Cruz, sc‐514302), anti‐Flag M2 (Sigma‐Aldrich, F1804), anti‐Myc 71D10 (Cell Signaling Technology, 2278), anti‐MEK2 13E3 (Cell Signaling Technology, 9147), anti‐phospho‐MEK1/2(S218/S222) 41G9 (Cell Signaling Technology, 9154), anti‐β‐actin 6D1 (Fujifilm Wako, Osaka, Japan, 010‐27841), anti‐caspase‐7 (Santa Cruz, sc‐81654), anti‐caspase‐8 1C12 (Cell Signaling Technology, 9746), anti‐caspase‐9 C9 (Cell Signaling Technology, 9508), anti‐RSK1/RSK2/RSK3 32D7 (Cell Signaling Technology, 9355); polyclonal anti‐MEK1 (Millipore, Billerica, MA, USA, 07‐641), anti‐B‐Raf (sc‐166), anti‐phospho‐ERK1/2 (Cell Signaling Technology, 9101), anti‐phospho‐MEK1(T292) (Chemicon, Temecula, CA, USA, AB4210), anti‐poly (ADP‐ribose) polymerase (PARP; Cell Signaling Technology, 9542), anti‐caspase‐3 (Cell Signaling Technology, 9662), anti‐caspase‐6 (Cell Signaling Technology, 9762), and anti‐phospho‐p90RSK(Thr573) (Cell Signaling Technology, 9346). All antibodies were used at a dilution of 1 : 1000. The following secondary antibodies were used at a dilution of 1 : 5000 (anti‐mouse Ab) or 1 : 2500 (anti‐rabbit Ab): anti‐mouse IgG‐horse radish peroxidase (HRP) antibody (NA931, Cytiva), and anti‐rabbit IgG‐HRP antibody (NA934, Cytiva).

### Immunoprecipitation and Phos‐tag SDS/PAGE

HEK293 cells were transfected with Myc‐MEK1, together with ERK2(PD) or Flag‐BRaf(V600E), and lysed in lysis buffer B. Cell lysates were incubated with anti‐Myc antibody at 4 °C for 3 h, and protein G‐sepharose beads (GE Healthcare) were then added and incubated for 1 h. Immunoprecipitates were collected by centrifugation and washed three times with lysis buffer B containing 0.1% SDS and separated on SDS/PAGE gels containing 50 μm phos‐tag acrylamide (Fujifilm Wako) and 0.1 mm MnCl_2_. After electrophoresis, gels were washed three times with transfer buffer (25 mm Tris, 192 mm glycine, 20% methanol) containing 10 mm EDTA, and then washed once with transfer buffer without EDTA according to the manufacturer's protocol. The separated proteins were transferred to a nitrocellulose membrane and probed with the appropriate antibodies.

### Detection of apoptotic cells

Apoptotic cells were detected using an annexin V‐ fluorescein isothiocyanate (FITC) Kit (MBL Life Science) according to the manufacturer's instructions. Briefly, cells were collected, resuspended in the binding buffer supplemented in the kit, and then incubated with annexin V‐FITC and Hoechst 33342 (Thermo Fisher, Waltham, MA, USA) in the dark for 20 min. Annexin V‐FITC‐bound cells stained in the plasma membrane were detected by fluorescence microscopy and counted. The experiments were carried out in triplicate.

### Statistical analysis

The statistical significance of the difference between mean values was tested using a two‐tailed Student's *t*‐test or one‐way ANOVA turkey test. Data are presented as mean ± SEM. A value of *P* < 0.05 was considered to indicate a statistically significant difference.

## Results

### MEK1 is cleaved in a caspase‐dependent manner during apoptosis

**Fig. 1:**
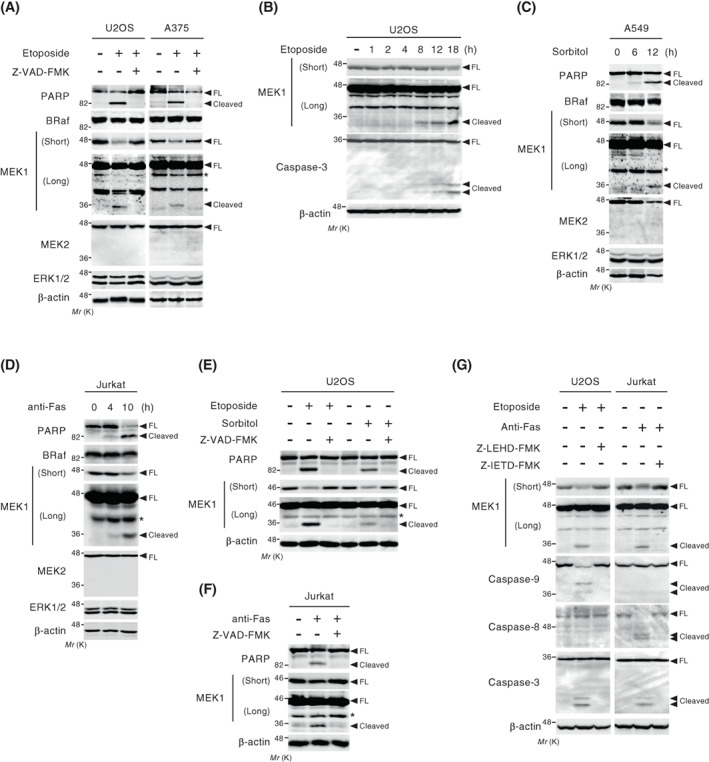
MEK1 is cleaved in a caspase‐dependent manner during apoptosis. (A) U2OS and A375 cells were treated with etoposide (60 μm for 24 h) and the expression level of endogenous MEK1 was analyzed by immunoblotting using an antibody against the N‐terminal region (amino‐acid residues 2–18) of MEK1. Etoposide induced the appearance of the cleaved 35‐kDa form of MEK1 (Cleaved). PARP, BRaf, MEK2, and ERK1/2 in cell extracts were also probed with the appropriate antibodies. Actin served as a loading control. Short, short exposure. Long, long exposure. FL, full‐length. *, nonspecific bands. (B) U2OS cells were stimulated with etoposide (60 μm) for the indicated times. The cleavage of MEK1 and PARP was analyzed by immunoblotting. (C, D) A549 (C) and Jurkat (D) cells were exposed to osmotic stress (0.6 m sorbitol) or anti‐Fas antibody (200 ng·mL^−1^) for the indicated times. Immunoblotting was performed as in (A). (E, F) U2OS (E) and Jurkat (F) cells were stimulated with etoposide (60 μm for 24 h), osmotic stress (0.6 m sorbitol for 6 h), or anti‐Fas antibody (200 ng·mL^−1^ for 8 h) as indicated. The induced proteolytic cleavage of PARP and MEK1 was analyzed by immunoblotting. (G) U2OS and Jurkat cells were stimulated with etoposide (60 μm for 24 h) or anti‐Fas antibody (200 ng·mL^−1^ for 10 h) as indicated. The cleavage of MEK1, caspase‐9, caspase‐8, and caspase‐3 was analyzed by immunoblotting using appropriate antibodies. In (A, E–G), cells were pretreated with (+) or without (−) a pan‐caspase inhibitor Z‐VAD‐FMK (100 μm), a caspase‐8 inhibitor Z‐IETD‐FMK (20 μm), or a caspase‐9 inhibitor Z‐LEHD‐FMK (20 μm) for 2 h before stimulation as indicated.

To examine whether the core components of the ERK pathway (i.e., Raf, MEK, and ERK) could be processed by caspases during apoptosis, we treated U2OS and A375 cells with a DNA‐damaging reagent, etoposide, and analyzed their protein expression levels by western blotting. As anticipated, etoposide treatment efficiently induced apoptotic cell death, as assessed by proteolytic cleavage of PARP, a prominent substrate of executioner caspases, and a well‐known marker of apoptosis (Fig. 1A). Following etoposide treatment, BRaf, MEK2, and ERK1/2 protein expression remained almost unchanged. In contrast, the expression level of full‐length MEK1 (an approximately 45‐kDa band) was markedly reduced in cells undergoing apoptosis. More importantly, concurrently with the reduction in full‐length MEK1 protein, a band of approximately 35 kDa appeared in the western blot using an antibody against the N‐terminal region (amino‐acid residues 2–18) of MEK1, suggesting that MEK1 is selectively cleaved to produce a 35‐kDa fragment during apoptosis. To examine whether this MEK1 cleavage occurs in a caspase‐dependent manner, U2OS and A375 cells were pretreated with a pan‐caspase inhibitor, Z‐VAD‐FMK, and then stimulated with etoposide. Inhibition of cellular caspase activities by Z‐VAD‐FMK completely abrogated the etoposide‐induced appearance of the 35‐kDa band and the reduction of full‐length MEK1. Furthermore, following etoposide treatment, the timing and amounts of the MEK1 cleavage coincided with those of caspase‐3 activation (Fig. 1B). Therefore, MEK1 is cleaved in a caspase‐dependent manner during apoptosis, resulting in the production of the 35‐kDa band.

Next, to test whether caspase‐dependent MEK1 cleavage takes place universally during apoptosis induced by various stimuli, we treated A549 and Jurkat cells with two distinct cell death‐inducing agents that activate the intrinsic (hyperosmotic stress; sorbitol) and extrinsic (an anti‐Fas antibody) apoptosis pathways, respectively (Fig. 1C,D). As with the case of etoposide treatment, these stimuli also reduced the expression level of full‐length MEK1, but not that of MEK2, and led to the appearance of the cleaved 35‐kDa fragment of MEK1. Again, this 35‐kDa band was abolished when the cells were pretreated with Z‐VAD‐FMK (Fig. 1E,F). Similarly, inhibition of etoposide‐ and Fas‐induced apoptosis at the level of the initiator caspases by their specific inhibitors (a caspase 9 inhibitor Z‐LEHD‐FMK for etoposide‐induced apoptosis and a caspase‐8 inhibitor Z‐IETD‐FMK for Fas‐induced apoptosis) also abolished the MEK1 cleavage (Fig. 1G). Thus, we concluded that, of the core components of the ERK pathway, MEK1 is the main target for caspase‐dependent proteolysis during apoptosis.

### MEK1 cleavage occurs at an evolutionarily conserved Asp282 residue

**Fig. 2:**
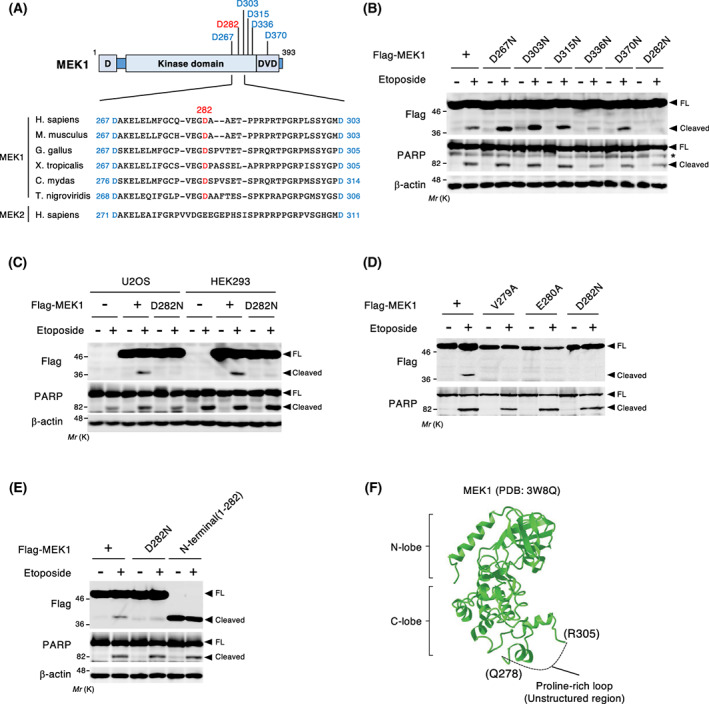
Caspase‐dependent MEK1 cleavage occurs at the Asp282 residue. (A) (Upper) Schematic diagram of potential caspase cleavage sites in MEK1. Six candidate cleavage sites predicted by CaspDB and ScreenCap3 databases are shown. D, the D‐site (ERK‐docking site). DVD, the domain for versatile docking site (MAPKKK‐docking site). (Lower) Sequence alignment of various MEK1 orthologs. MEK1 cleavage sites are shown in red. The amino acid sequence of human MEK2 is also shown. (B, D, E) MEK1 is cleaved at Asp282 during apoptosis. U2OS cells were transiently transfected with wild‐type (+), point‐mutated, or C‐terminally truncated (a.a. 1–282) forms of Flag‐MEK1 as indicated, and treated with etoposide (60 μm for 24 h). The cleavage of Flag‐MEK1 and endogenous PARP was assessed by immunoblotting using appropriate antibodies as indicated. (C) U2OS cells stably expressing Flag‐MEK1 [wild‐type (+) or D282N mutant] were treated with etoposide, and MEK1 cleavage was assessed as in (B). PARP and β‐actin (a loading control) were also probed as indicated. (F) The three‐dimensional structure of human MEK1 (PDB: 3W8Q; https://www.rcsb.org/structure/3w8q). The dotted line indicates the unstructured proline‐rich loop region, which is exposed on the surface of the kinase C‐lobe and contains the cleavage site (Asp282).

We next sought to identify the cleavage site on MEK1. Because, in general, caspases cleave their substrate proteins after an Asp residue within a specific tetrapeptide motif (xxxD) [29], and because the cleaved, N‐terminal fragment of MEK1 was approximately 35 kDa in size, we focused on Asp residues located in the C‐terminal half of the MEK1 protein sequence. *In silico* analyses using two caspase cleavage site prediction tools (CaspDB and ScreenCap3) predicted a total of six candidate sites in MEK1 (Fig. 2A). We then generated a series of MEK1 point mutants, in which each of the predicted Asp residues was substituted with an Asn residue (Fig. 2B). These mutants were then transiently transfected into U2OS cells, and their etoposide‐induced, caspase‐mediated cleavage was monitored by immunoblotting. Although etoposide treatment robustly induced the cleavage of five of six MEK1 mutants, no cleavage was observed in the MEK1(D282N) mutant. Virtually identical results were also obtained when MEK1(D282N) was stably expressed in U2OS and HEK293 cells (Fig. 2C), suggesting that the Asp282 residue is the site for caspase‐mediated cleavage of MEK1. To further confirm caspase‐dependent MEK1 cleavage at Asp282, we introduced point mutations near this site (i.e., V279A and E280A) since caspases recognize a tetrapeptide motif within a substrate protein for cleavage. As anticipated, both mutants lost their ability to be cleaved during apoptosis (Fig. 2D). Finally, we found that an artificially synthesized, N‐terminal fragment of MEK1 (a.a. 1–282) exhibited a similar electrophoretic mobility by SDS/PAGE to the caspase‐dependent cleaved form of endogenous MEK1 (Fig. 2E). Thus, we concluded that MEK1 is cleaved at Asp282 by a caspase(s) *in vivo*. Since Asp282 is located within the flexible proline‐rich loop of MEK1 and is exposed on the surface of the kinase domain (Fig. 2F), this site is likely to be accessible for caspase‐mediated proteolysis. Furthermore, Asp282 and its surrounding residues are highly conserved among MEK1, but not MEK2, orthologs in diverse organisms (Fig. 2A), suggesting that caspase‐mediated MEK1 cleavage during apoptosis might be similarly conserved.

### MEK1 is specifically cleaved by caspase‐3

**Fig. 3:**
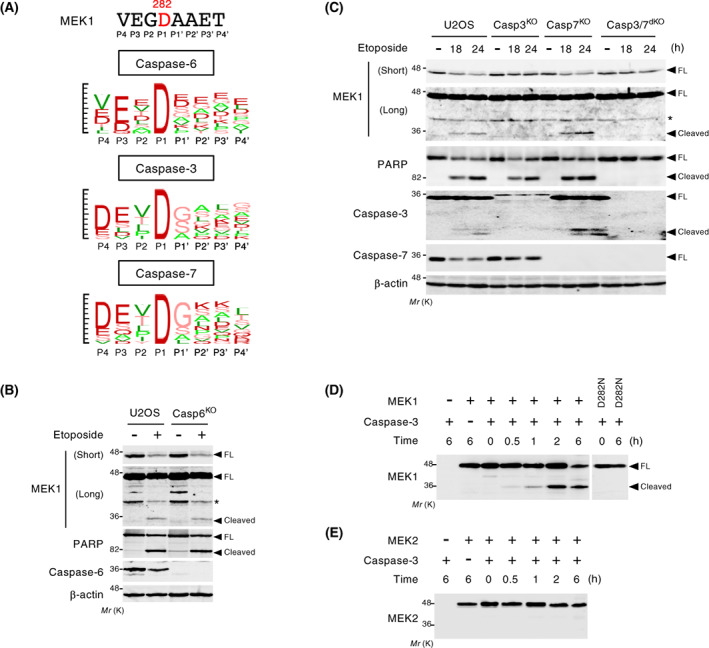
MEK1 is specifically cleaved by caspase‐3. (A) Sequence logo representations of the occurrences of amino acid residues in cleavage sites (P4–P4′ positions) of human caspase‐6/3/7. Logos were generated using a SitePrediction web tool (https://www.dmbr.ugent.be/prx/bioit2‐public/SitePrediction/index.php). The cleavage site sequences (P4–P4′) of known substrates for human caspase‐6/3/7 were obtained from a MEROPS database (https://www.ebi.ac.uk/merops/). (B) Parental U2OS and Casp6^KO^ cells were treated with etoposide (60 μm for 24 h), and MEK1 cleavage was assessed by immunoblotting using an anti‐MEK1 antibody. (C) Parental U2OS, Casp3^KO^, Casp7^KO^, and Casp3/7^dKO^ cells were treated with etoposide (60 μm) for the indicated times, and MEK1 cleavage was assessed as in (B). (B, C) Expression levels of endogenous PARP and caspase‐6/3/7 were analyzed by immunoblotting. β‐actin served as a loading control. (D, E) *In vitro* cleavage assay of purified recombinant caspase‐3. Bacterially expressed, recombinant MEK1(WT or D282N) (D) or MEK2 (E) was incubated with caspase‐3 for the indicated times *in vitro*. The reaction mixture was separated by SDS/PAGE, and probed with an anti‐MEK1 (D) or an anti‐MEK2 (E) antibody.

To identify the caspase(s) responsible for the cleavage of MEK1, we initially compared the amino acid sequence surrounding the MEK1 cleavage site (VEGD^282^) with previously reported consensus recognition motifs of individual executioner caspases [30]. Consequently, the sequence of the MEK1 cleavage site appeared most similar to the consensus cleavage motif for caspase‐6 among those of executioner caspases (caspase‐3/6/7) (Fig. 3A). Therefore, we first tested whether caspase‐6 is involved in MEK1 cleavage *in vivo*. For this purpose, we generated caspase‐6‐null U2OS cells (termed Casp6^KO^ cells) using CRISPR‐Cas9 technology and examined whether MEK1 cleavage was suppressed in these cells. Etoposide treatment, however, induced MEK1 cleavage in Casp6^KO^ cells as efficiently as that in control U2OS cells, suggesting that caspase‐6 was not responsible for MEK1 cleavage (Fig. 3B). Next, to assess the involvement of the other executioner caspases, we again used CRISPR‐Cas9 to generate caspase‐3, or/and caspase‐7 knockout cells (hereafter called Casp3^KO^, Casp7^KO^, Casp3/7^dKO^, respectively). As shown in Fig. 3C, in Casp3/7^dKO^ cells, reflecting the lack of these two major executioner caspases, etoposide treatment failed to induce PARP cleavage, and therefore MEK1 cleavage was also completely abrogated. In contrast, etoposide treatment efficiently induced PARP cleavage in both Casp3^KO^ and Casp7^KO^ cells at a level comparable to that observed in parental U2OS cells, indicating a redundant role for caspase‐3 and ‐7 in the proteolysis of PARP. Interestingly, however, MEK1 cleavage was completely abolished in Casp3^KO^ cells, whereas no inhibition of MEK1 cleavage was observed in Casp7^KO^ cells. Thus, caspase‐3, but not caspase‐7, is a prerequisite for proteolytic cleavage of MEK1 *in vivo*.

To test directly whether caspase‐3 can cleave MEK1 at Asp282, but not MEK2, we performed *in vitro* cleavage assays of bacterially expressed, purified caspase‐3 using recombinant MEK1, MEK1(D282N), and MEK2 proteins as substrates. As shown in Fig. 3D,E, caspase‐3 directly cleaved MEK1, but not MEK1(D282N) or MEK2. We concluded from the combined data that MEK1 is a highly specific substrate for caspase‐3 and is thus cleaved by the caspase at the evolutionarily conserved Asp282 residue during apoptosis.

### Phosphorylation states of MEK1 do not affect caspase‐mediated cleavage

**Fig. 4:**
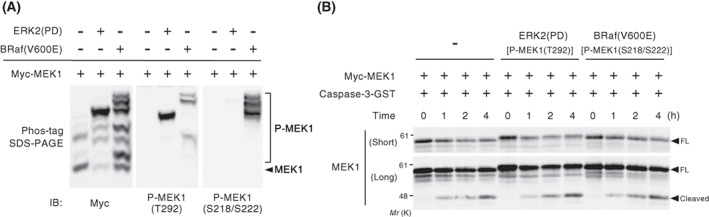
Caspase‐3 can cleave MEK1 irrespective of its phosphorylation states. (A) HEK293 cells were transfected with Myc‐MEK1, together with active ERK2(PD) or active BRaf(V600E) mutants. Immunopurified Myc‐MEK1 was separated by Phos‐tag SDS/PAGE and immunoblotted with antibodies specific for Myc (left), phospho‐MEK1(T292) (middle), or phospho‐MEK1 (S218/S222) (right) to assess its phosphorylation states. (B) *In vitro* cleavage assay of purified recombinant caspase‐3. The phosphorylated forms of Myc‐MEK1 immunopurified from HEK293 cells co‐expressing ERK2(PD) or BRaf(V600E) were incubated with caspase‐3 for the indicated times. MEK1 cleavage was monitored by immunoblotting using an anti‐Myc antibody.

The kinase activity of MEK1 is controlled by multiple phosphorylation events. Upon growth factor stimulation, Raf activates MEK1 by phosphorylating Ser218 and Ser222 within its T‐loop, while activated ERK also phosphorylates MEK1 at Thr292 in feedback phosphorylation and attenuates its kinase activity to some extent [1]. Therefore, we next tested whether such phosphorylation events affected caspase‐mediated MEK1 cleavage. To induce the phosphorylation of MEK1, we transfected HEK293 cells with Myc‐tagged MEK1, together with active BRaf(V600E) (for preferentially phosphorylating S218/S222) or active ERK2(PD) (for preferentially phosphorylating T292) mutants. We then assessed the phosphorylation states of MEK1 at S218/S222 and at T292 using a Phos‐tag SDS/PAGE technique, which leads to retarded electrophoretic mobility of phosphorylated proteins and thus visualizes these as up‐shifted bands. Immunopurified Myc‐MEK1 proteins were separated by Phos‐tag SDS/PAGE, and the phosphorylation states of Myc‐MEK1 at S218/S222 or T292 were analyzed by immunoblotting with anti‐Myc, anti‐phospho‐MEK1(S218/S222), or anti‐phospho‐MEK1(T292) antibodies. Co‐expression of ERK2(PD) or BRaf(V600E) induced multiple slower‐migrating Myc‐MEK1 bands, which corresponded to MEK1 phosphorylation at S218/S222, T292, and/or other sites (Fig. 4A). Densitometric analyses of these bands revealed that more than 90% of Myc‐MEK1 protein was phosphorylated at Thr292 when co‐expressed with ERK2(PD), and approximately 60% at S218/S222 when co‐expressed with B‐Raf(V600E). We then immunopurified these phosphorylated proteins from cell extracts and incubated them with recombinant caspase‐3 *in vitro*. As shown in Fig. 4B, *in vitro* cleavage assays showed that Myc‐MEK1 proteins preferentially phosphorylated at T292 or at S218/S222 were cleaved by caspase‐3 as efficiently as unphosphorylated MEK1. Thus, these findings strongly suggest that caspase‐3 can cleave MEK1 during apoptosis irrespective of its phosphorylation (activation) states.

### Caspase‐3‐mediated cleavage of MEK1 abrogates its kinase activity

**Fig. 5:**
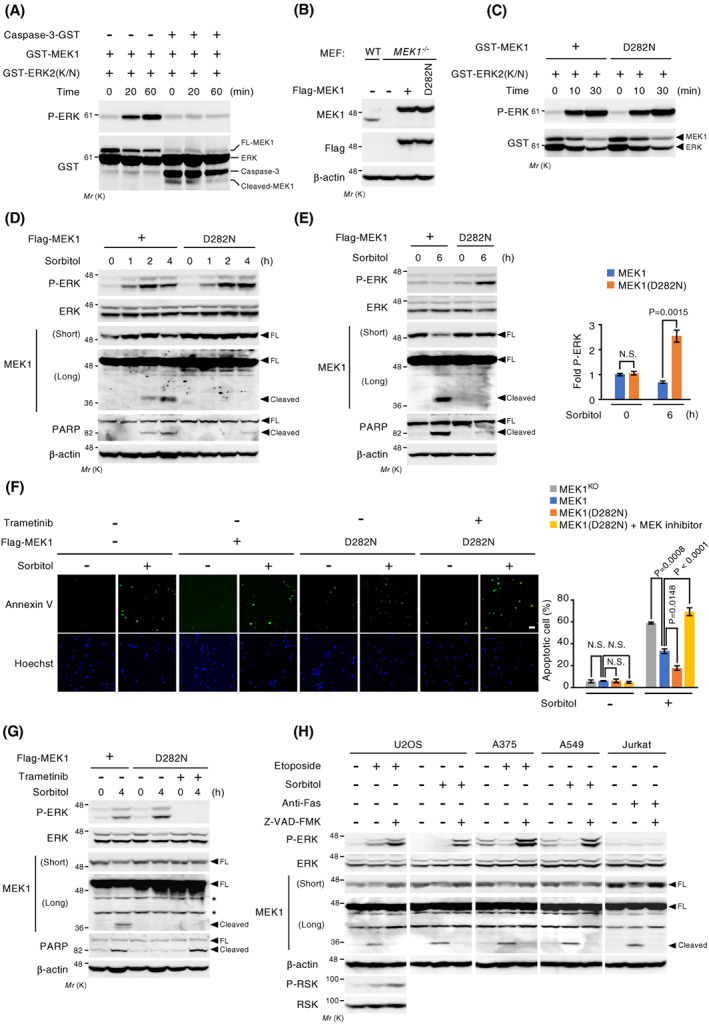
MEK1 cleavage by caspase‐3 abolishes its kinase activity and promotes apoptosis. (A) *In vitro* kinase assay of full‐length or caspase‐3‐mediated cleaved forms of GST‐MEK1. GST‐MEK1 was first incubated with or without purified, recombinant caspase‐3‐GST, and was then mixed and further incubated with a MEK1 substrate, kinase‐defective GST‐ERK2(K/N), for the indicated times. Phosphorylated ERK2(K/N) was detected by immunoblotting (top). Total GST‐ERK2(K/N) and GST‐MEK1 (full‐length or cleaved forms) were also probed with an anti‐GST antibody (bottom). (B) Expression levels of endogenous MEK1 and exogenous Flag‐MEK1 (WT or D282N) in wild‐type MEF, *MEK1* ^*−/−*^ MEF, MEF‐MEK1, and MEF‐MEK1(D283N) cells were analyzed by immunoblotting. (C) *In vitro* kinase assay of recombinant GST‐MEK1 or GST‐MEK1(D282N). The assay was performed as in (A). (D, E) MEF‐MEK1 or MEF‐MEK1(D282N) cells were exposed to osmotic stress (0.6 m sorbitol) for the indicated times. The phosphorylation status of ERK, and the cleavage of MEK1 and PARP were analyzed by immunoblotting. In (E), the intensity of the P‐ERK bands from three independent experiments was quantified (right graph). Data are mean ± SEM. *P*‐values were assessed using a two‐tailed Student's *t*‐test. N.S., not significant. (F) *MEK1* ^*−/−*^MEF, MEF‐MEK1, and MEF‐MEK1(D282N) cells were treated with osmotic stress (0.6 m sorbitol for 3 h), stained with annexin V‐FITC, and visualized by fluorescence microscopy (upper panels). The scale bar represents 50 μm. The percentage of annexin V‐positive apoptotic cells was quantified (right graph). Data are mean ± SEM from three independent experiments. More than 100 cells were counted per sample. *P*‐values were assessed using one‐way ANOVA followed by Tukey's multiple comparisons test. N.S., not significant. (G) The cells were exposed to osmotic stress (0.6 m sorbitol for 4 h) in the presence or absence of a MEK inhibitor (10 μm trametinib). The phosphorylation status of ERK, and the cleavage of MEK1 and PARP were analyzed by immunoblotting. (H) The indicated cells were stimulated with etoposide (60 μm for 24 h), sorbitol (0.6 m for 12 h), or anti‐Fas antibody (200 ng·mL^−1^ for 10 h) as indicated. Where indicated, the cells were pretreated with (+) or without (−) the pan‐caspase inhibitor Z‐VAD‐FMK. The phosphorylation states of ERK and RSK, and the cleavage of MEK1 were monitored by immunoblotting. (B, D, E, G, H) Actin served as a loading control.

Since the MEK1 cleavage site (Asp282) is located within its kinase domain, we asked whether caspase‐mediated MEK1 cleavage affected its enzymatic activity. For this purpose, we measured the kinase activity of the cleaved form of MEK1 *in vitro* (Fig. 5A). Recombinant GST‐MEK1 was first incubated with purified caspase‐3 to achieve its cleavage at Asp282, and then activity of the cleaved MEK1 was assessed by an *in vitro* kinase assay using a kinase‐defective GST‐ERK2(K52N) as a substrate. As expected, control (full‐length) MEK1 efficiently phosphorylated GST‐ERK2(K52N) over time. In contrast, the cleaved MEK1 completely lost its ability to phosphorylate GST‐ERK2(K52N). Thus, the caspase‐mediated cleavage of MEK1 abolished its kinase activity.

### Cleavage of MEK1 promotes apoptosis

Since the ERK pathway inhibits apoptotic cell death through several different mechanisms and MEK1 has been reported to be more important than MEK2 for cell growth and survival [18, 19, 31], we next tested whether caspase‐dependent MEK1 cleavage and resulting MEK1 inhibition accelerated apoptotic cell death. For this purpose, we established *MEK1* ^*−/−*^ MEFs re‐expressing either wild‐type MEK1 or the caspase‐uncleavable MEK1(D282N) mutant [termed MEF‐MEK1 and MEF‐MEK1(D282N) cells, respectively] (Fig. 5B). Initially, we determined that the D282N mutation *per se* did not affect MEK1 kinase activity. Both MEK1 and MEK1(D282N) phosphorylated GST‐ERK2(K/N) to a similar extent in an *in vitro* kinase assay (Fig. 5C). We then monitored the sensitivity of these two cell lines to osmotic stress since this induces not only apoptosis but also ERK activation. Following sorbitol treatment, wild‐type MEK1 was gradually cleaved (inactivated) with time, while the caspase‐uncleavable MEK1(D282N) remained intact throughout the experimental period (Fig. 5D,E). Accordingly, MEF‐MEK1(D282N) cells exhibited stronger ERK activity than MEF‐MEK1 cells particularly in the late phase of ERK activation after osmotic stress exposure during which wild‐type MEK1 was inactivated by caspase‐dependent proteolysis. Moreover, consistent with the higher activity of pro‐survival ERK signaling, MEF‐MEK1(D282N) cells showed substantially reduced apoptosis when compared with control MEF‐MEK1 cells, as assessed by the cleavage of PARP. Similar results were also obtained when apoptotic cell death was monitored by annexin V staining assay (Fig. 5F). After osmotic stress, the percentage of annexin V‐positive apoptotic cells was significantly decreased in MEF‐MEK1(D282N) cells, compared with MEF‐MEK1 cells. Suppression of cellular ERK activity by a MEK inhibitor, trametinib, in MEF‐MEK1(D282N) cells restored osmotic stress‐induced apoptosis, as assessed by Annexin V staining (Fig. 5F) and PARP cleavage (Fig. 5G). Thus, the caspase‐mediated cleavage of MEK1 suppresses pro‐survival ERK signaling, thereby promoting apoptotic cell death. We confirmed that caspase‐dependent MEK1 cleavage and the resulting ERK inhibition were also observed in several other cell types (i.e., U2OS, A375, A549, and Jurkat cells) under various stress conditions (Fig. 5H).

### A RASopathy‐associated MEK1(Y130C) mutation abrogates MEK1 cleavage

**Fig. 6:**
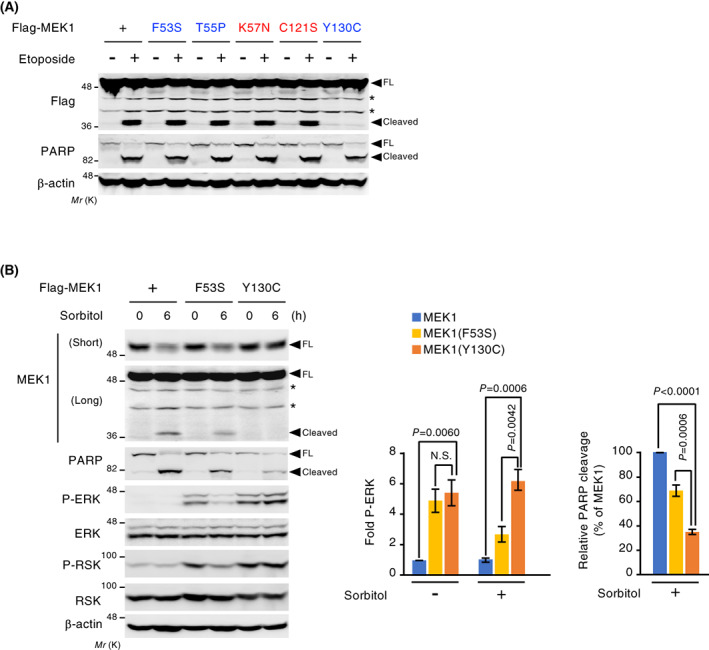
MEK1(Y130C) mutation abrogates MEK1 cleavage and efficiently suppresses apoptosis. (A) U2OS cells were transiently transfected with Flag‐MEK1 or its mutant derivatives. RASopathy‐ and cancer‐associated mutants are shown in blue and red, respectively. After etoposide treatment (60 μm for 24 h), the cleavage of Flag‐MEK1 and endogenous PARP was assessed by immunoblotting using total cell extracts. *, nonspecific bands. (B) MEF‐MEK1, MEF‐MEK1(F53S), or MEF‐MEK1(Y130C) cells were treated with sorbitol (0.6 m) for the indicated times. The cleavage of MEK1 and PARP, and the phosphorylation states of ERK and RSK were analyzed by immunoblotting using appropriate antibodies. The intensity of the P‐ERK and cleaved PARP bands from three independent experiments was quantified (right graphs). Data are mean ± SEM. *P*‐values were assessed using one‐way ANOVA followed by Tukey's multiple comparisons test. N.S., not significant.

Various point mutations in the *MEK1* gene have been reported in congenital RASopathies as well as sporadic human cancers [23, 24]. Since previous studies regarding disease‐associated MEK1 mutants have mainly focused on their kinase activity, the effects of the mutations on other properties of the MEK1 protein remain unknown. Therefore, we examined whether the disease‐associated mutations affected the caspase‐mediated regulation of MEK1. For this purpose, U2OS cells were transiently transfected with representative disease‐associated MEK1 mutants (F53S, T55P, and Y130C for RASopathies, and K57N and C121S for cancers) and were then treated with etoposide and analyzed for MEK1 cleavage by immunoblotting (Fig. 6A). Interestingly, we found that, although most of the mutants tested were robustly cleaved during apoptosis, one of the mutants, the RASopathy‐derived MEK1(Y130C), was completely resistant to cleavage, indicating that Y130C mutation prevents MEK1 from caspase‐dependent proteolytic inhibition.

Next, to compare the effects of the caspase‐cleavable MEK1(F53S) and caspase‐noncleavable MEK1(Y130C) mutants on stress‐induced apoptotic cell death, we established *MEK1* ^*−/−*^ MEFs stably re‐expressing either Flag‐MEK1(F53S) or Flag‐MEK1(Y130C) [termed MEF‐MEK1(F53S) and MEF‐MEK1(Y130C) cells, respectively]. These cells were treated with osmotic stress, and the phosphorylation states of ERK and RSK (an ERK substrate) as well as the cleavage of MEK1 and PARP were assessed by immunoblotting (Fig. 6B). Consistent with a previous report [23], MEK1(F53S) and MEK1(Y130C) mutants showed a comparable level of elevated basal kinase activity in a steady state, as assessed by phosphorylation states of ERK and RSK. When these cells were exposed to osmotic stress, wild‐type MEK1 and MEK1(F53S) were efficiently cleaved (inactivated), while MEK1(Y130C) remained intact even 6 h after osmotic stress exposure. Accordingly, MEF‐MEK1(Y130C) cells exhibited stronger phosphorylation of ERK and RSK than MEF‐MEK1 and MEF‐MEK1(F53S) cells under the stress conditions. Moreover, consistent with the higher ERK activity, MEF‐MEK1(Y130C) cells showed significantly reduced apoptosis when compared with MEF‐MEK1 or MEF‐MEK1(F53S) cells, as assessed by the cleavage of PARP. These findings indicate that the MEK1(Y130C) mutant efficiently suppresses apoptotic cell death not only by its high basal kinase activity but also by escaping from caspase‐mediated inhibition.

## Discussion

In this study, we identified MEK1, a direct activator of ERK, as a highly specific substrate for executioner caspase‐3, and demonstrated that caspase‐3‐mediated MEK1 cleavage suppressed pro‐survival ERK signaling, thereby sensitizing cells to apoptotic cell death. While the importance of protein phosphorylation‐dephosphorylation events in the regulation of the ERK cascade is well‐documented, less is known about the roles of other posttranslational modifications in this signaling. We found that, of the core components of the ERK pathway (i.e., Raf, MEK, and ERK), the expression level of only MEK1 was substantially decreased by caspase‐dependent proteolysis during apoptosis, and that this MEK1 cleavage occurred irrespective of its phosphorylation status and abolished its kinase activity. Furthermore, since the 35‐kDa fragment of cleaved MEK1 contains the intact ERK‐docking site, this inactive MEK1 fragment may act as a dominant negative inhibitor for ERK signaling by interacting with and sequestering ERK in cells undergoing apoptosis. Therefore, our data indicate that, during apoptosis, the ERK pathway is downregulated by caspase‐3‐dependent cleavage of MEK1 to further promote apoptotic cell death.

Our *in vitro* and *in vivo* experiments showed that caspase‐3 efficiently cleaved MEK1 at the Asp282 residue, which is located within the proline‐rich loop of its kinase domain. Consistent with the fact that this loop region of MEK1 is divergent in sequence from that of its paralog MEK2, MEK1, but not MEK2, was selectively cleaved during apoptosis. Although further studies are required to comprehensively elucidate the roles of MEK1‐specific proteolysis, previous studies have shown that MEK1 and MEK2 have not only overlapping but also unique biological functions. For instance, mouse embryos deficient in the *MEK1* gene are prenatally lethal due to placental abnormalities [32], whereas *MEK2* mutant mice are viable and fertile [33]. In various cell lines, depletion of MEK1 by short hairpin RNA showed a marked inhibition of cell growth and survival, whereas MEK2 depletion exhibited less pronounced inhibitory effects [18, 19, 31]. Consistently, MEK1 is much more efficiently activated by serum than MEK2 [17]. Furthermore, in a mouse skin carcinogenesis model, 7,12‐dimethylbenz[a]anthracene (DMBA)/12‐O‐tetradecanoylphorbol‐13‐acetate (TPA)‐induced tumor formation was profoundly suppressed in *MEK1*‐deficient mice as compared with wild‐type mice, whereas no obvious inhibitory effect was observed in *MEK2*‐deficient mice [34]. Altogether, these findings suggest that MEK1 is more important than MEK2 for cell growth, survival, embryonic development, and carcinogenesis. Thus, MEK1‐specific inhibition by caspase‐mediated cleavage may suffice to promote apoptotic cell death under stress conditions (e.g., osmotic stress, DNA‐damage, and Fas stimulation) as we observed in the present study, and thus to support the maintenance of biological homeostasis. Since the Asp282 residue is highly conserved among vertebrates, caspase‐3‐mediated cleavage of MEK1 during apoptosis might be similarly conserved across species.

Previous studies showed that, although capsase‐6, a divergent member of the executioner caspase family, is unique in its substrate profile, the two major executioner caspases, caspase‐3 and ‐7, share similar substrate specificities in *in vitro* cleavage assays using positional scanning synthetic peptide libraries [29, 35]. Therefore, caspase‐3 and ‐7 were initially considered functionally redundant proteases. More recent studies, however, revealed that these two caspases possess differential activity toward multiple substrate proteins and thus can play distinct roles during apoptosis. Indeed, caspase‐3 has been shown to have a broader substrate spectrum than caspase‐7. Caspase‐3, but not caspase‐7, efficiently cleaves XIAP, gelsolin, Bid, and procaspase‐9 *in vitro* [36, 37]. Immuno‐depletion of caspase‐3 from Jurkat cell‐free extracts abolished cytochrome *c*/dATP‐inducible cleavage of various caspase substrates, whereas that of caspase‐7 had little impact on the same panel of caspase substrates [38]. Furthermore, *in vivo* studies using genetically engineered mice showed that, although caspase‐7‐knockout mice were viable, caspase‐3‐deficient 129X1/SvJ mice died perinatally of brain hyperplasia caused by decreased apoptosis of neurons and glial cells [39, 40]. Thus, caspase‐3 has more significant roles than caspase‐7 during the demolition phase of apoptosis. In the present study, by analysis of caspase‐3, ‐6 or/and ‐7 knockout cells, we found that MEK1 is a specific substrate for caspase‐3 and that its caspase‐3‐dependent cleavage attenuated pro‐survival ERK signaling, thereby accelerating apoptotic cell death. Therefore, our findings may at least partially explain the striking destructive feature of caspase‐3 in the apoptotic process.

Another interesting finding of the present study is that the RASopathy‐associated Y130C mutation prevents the caspase‐3‐dependent, proteolytic inhibition of MEK1. In patients with RASopathies, particularly in CFC syndrome, more than 10 germline missense‐mutations in MEK1 have been reported [41, 42]. Interestingly, among MEK1/2 mutations detected in RASopathies, MEK1(Y130C) is the most prevalent genetic alteration and accounts for more than 40% of all MEK mutation‐positive cases. Although these RASopathy‐associated MEK1 mutants have been shown to have moderately increased kinase activity [23], the detailed biological properties and dysregulation of individual mutants are, however, still obscure. In this study, we revealed that the Y130C mutation rendered MEK1 resistant to caspase‐3‐mediated cleavage. Therefore, our results suggest that MEK1(Y130C) remains intact and catalytically active under even harsh conditions where caspases can be activated (e.g., stress and death receptor activation), and suppresses apoptosis by maintaining highly active pro‐survival ERK signaling in cells. Indeed, MEF‐MEK1(Y130C) cells showed increased ERK activity and reduced apoptosis under osmotic stress conditions, as compared with MEF‐MEK1 or MEF‐MEK1(F53S) cells. Since rigorous control of apoptosis and the ERK pathway are both essential for normal embryonic development and homeostasis, the MEK1(Y130C) mutation may more severely perturb these biological processes than other types of MEK1 mutants. Indeed, a recent multinational cohort study of 138 individuals with CFC syndrome indicated that patients with a MEK1(Y130C) mutation exhibited a considerably higher prevalence and severity of neurologic symptoms, including treatment‐resistant severe epilepsy, than those with other MEK mutations [43]. Consistently, knock‐in mice harboring the MEK1(Y130C) mutation exhibited not only cardiac and craniofacial abnormalities but also marked neuropathological changes such as an increased number of astrocytes and oligodendrocyte precursors in the brain [44]. Thus, the disruption of caspase‐3‐dependent MEK1 inhibition by the Y130C mutation may lead to severe developmental abnormalities and thereby manifest as overt clinical symptoms in patients with a RASopathy.

In conclusion, we identified MEK1 as a highly specific substrate for caspase‐3. During apoptosis, MEK1 is cleaved at Asp282, thereby losing its kinase activity. This caspase‐3‐mediated, proteolytic inactivation of MEK1 sensitizes cells to apoptosis by attenuating pro‐survival ERK signaling. Our results demonstrate that MEK1 cleavage mediates the functional crosstalk between protective (pro‐survival ERK) and destructive (pro‐apoptotic caspase) signaling pathways, and delineate a molecular mechanism that regulates cell‐fate decisions under stress. Since this mechanism is disrupted by a RASopathy‐associated MEK1(Y130C) mutation, its dysregulation may be involved in the pathophysiology of RASopathies.

## Conflict of interest

The authors declare no conflict of interest.

## Peer review

The peer review history for this article is available at https://publons.com/publon/10.1002/2211‐5463.13574.

## Authors contributions

HM, YK, TT, and RN conducted the experiments. HM, YK, and MT designed the experiments and analyzed the data. HM, YK, and MT wrote the paper.

## Data accessibility section

The data that support the findings of this study are available from the corresponding author (takekawa@ims.u-tokyo.ac.jp) upon reasonable request.
